# High Israeli mortality rates from diabetes and renal failure - Can international comparison of multiple causes of death reflect differences in choice of underlying cause?

**DOI:** 10.1186/s13584-015-0027-6

**Published:** 2015-10-01

**Authors:** Nehama Goldberger, Yael Applbaum, Jill Meron, Ziona Haklai

**Affiliations:** Division of Health Information, Ministry of Health, 39 Yirmiyahu Street, 9101002 Jerusalem, Israel

**Keywords:** Diabetes, Kidney disease, Renal failure, Multiple causes of death, Choosing underlying cause of death, IRIS

## Abstract

**Background:**

The age-adjusted mortality rate in Israel is low compared to most Western countries although mortality rates from diabetes and renal failure in Israel are amongst the highest, while those from cardiovascular diseases (CVD) are amongst the lowest. This study aims to assess validity of choice of underlying causes (UC) in Israel by analyzing Israeli and international data on the prevalence of these diseases as multiple causes of death (MCOD) compared to UC, and data on comorbidity (MCOD based).

**Methods:**

Age-adjusted death rates were calculated for UC and MCOD and the corresponding ratio of multiple to underlying cause of death (SRMU) for available years between 1999 and 2012. Comorbidity was explored by calculating cause of death association indicators (CDAI) and frequency of comorbid disease. These results were compared to data from USA, France, Italy, Australia and the Czech Republic for 2009 or other available year.

**Results:**

Mortality rates for all these diseases except renal failure have decreased in Israel between 1999 and 2012 as UC and MCOD. In 2009, the SRMU for diabetes was 2.7, slightly lower than other Western countries (3.0–3.5) showing more frequent choice as UC. Similar results were found for renal failure. In contrast, the SRMU for ischemic heart disease (IHD) and cerebrovascular disease were 2.0 and 2.6, respectively, higher than other countries (1.4–1.6 and 1.7–1.9, respectively), showing less frequent choice as UC. CDAI data showed a strong association between heart and cerebrovascular disease, and diabetes in all countries. In Israel, 40 % of deaths with UC diabetes had IHD and 24 % had cerebrovascular disease. Renal disease was less strongly associated with IHD.

**Conclusion:**

This international comparison suggests that diabetes and renal failure may be coded more frequently in Israel as UC, sometimes instead of heart and cerebrovascular disease. Even with some changes in coding, mortality rates would be high compared to other countries, similar to the comparatively high diabetes prevalence in Israel at older ages and high rate of end-stage renal failure.

This study highlights the importance of physician training on death certification practice and need for further progress towards automation in recording and coding death causes.

**Electronic supplementary material:**

The online version of this article (doi:10.1186/s13584-015-0027-6) contains supplementary material, which is available to authorized users.

## Background

The age standardized mortality rate from all causes of deaths is low in Israel compared to most OECD countries [1], but the corresponding rates for deaths from diabetes and kidney disease as underlying cause (UC) are amongst the highest. In contrast, mortality rates from cardiovascular diseases (CVD), heart diseases and cerebrovascular disease are low compared to other countries [1].

In accordance with international death certification guidelines, the notification of death form in Israel includes the immediate cause of death, the train of events that led to this cause and other significant diseases, conditions or injuries that contributed to death but were not directly related to the disease or condition causing death. The Central Bureau of Statistics (CBS) chooses the UC according to definitions and conventions of the International Classification of Diseases (ICD) and has coded it according to ICD-10 since 1998. Choice of UC is manual, although in recent years occasional use has been made of Iris, an automatic system for coding multiple causes of death and selection of the UC, in particular in cases where the UC is unclear. In addition, in 1999, 2003 and since 2007, all causes mentioned on the notification of death form (multiple causes of death, MCOD) have been coded by the CBS.

In many other countries, too, data on MCOD have become available and analyzed in recent years. Redelings et al. [2] published a comparison of underlying and multiple cause mortality in the USA in 2000–2001. A French and Italian group of researchers, Desesquelles et al. [3], developed two standard indicators which can be used to analyze MCOD data and the mortality profiles they present. They used these indicators to explore mortality patterns in France and Italy, and published tables and graphic representations. They also initiated a collection of internationally comparable data for these indicators, the Multicause Network [4], and workshops to discuss use of MCOD data.

The Australian Institute of Health and Welfare also published a detailed analysis of MCOD for chronic diseases [5]. The USA has data available on MCOD from the Centers for Disease Control and Prevention (CDC) on the WONDER database [6]. Amongst other recent studies using MCOD data were those estimating the high burden of sepsis related mortality in England [7], and the USA [8], and of diabetes in France [9] and Finland [10]. Lu et al. [11] compared diabetes mortality rates as UC and MCOD, and frequency of choice of diabetes as UC and of diabetes in part 1 of the death certificate, from all diabetes reported deaths, in Taiwan, Australia and Sweden.

The problem of choosing diabetes as UC, in particular when coexisting CVD are found, has also been raised by Adair and Rao [12] who found an increasing ratio of diabetes with CVD in part I of the death certificate compared to part II, in Australia, and to a lesser extent in the USA, between 1999 and 2006. They suggest that this may affect choice of UC and contribute to increasing diabetes mortality rates, and point to the need for international guidelines on certifying deaths with diabetes and CVD. Nojilana et al. [13] also wrote of the need for such guidelines in view of wide country variations in the proportion of diabetes related deaths with diabetes mentioned in part I of the death certificate.

This study presents recent mortality rates in Israel for diabetes, renal failure and cardiovascular diseases as UC and as MCOD. The study then investigates whether analysis of Israeli and international data on disease prevalence found in MCOD compared to UC, and on comorbidity from MCOD can validate the choice of UC in Israel. Are the extremely high and low values due to coding practices, such as choosing diabetes and kidney disease instead of CVD as UC of death?

## Methods

Israeli mortality data on underlying, and all contributory causes of death was taken from the nationwide database of causes of death prepared by the Central Bureau of Statistics (CBS) with causes coded according to ICD-10. MCOD data was available for the years 1999, 2003 and 2007–2012.

Desesquelles et al. [3], defined two standard indicators: 1. The Standardized Ratio of Multiple to Underlying cause (SRMU), which is the ratio of the age standardized mortality rate for any mention of a cause (from MCOD), divided by the age standardized mortality rate for the cause when chosen as underlying cause (UC). This ratio reflects the frequency of mention of the cause on the death certificate compared to its choice as underlying cause, and is low for conditions usually selected as underlying and high for those rarely chosen. 2. The Cause of Death Association Indicator (CDAI), which is a ratio of the proportion of a contributory cause among all deaths chosen with a specific underlying cause to its proportion among all deaths. The calculation of this indicator uses standard death counts to enable international comparison (see formula in Table 2). This indicator represents the frequency of occurrence of underlying and contributory causes together, and when significantly greater than 100 (with a 95 % confidence interval over 100), shows a greater association than would be expected randomly.

Age standardized mortality rates in Israel were calculated for the UC and MCOD, and the ratio of multiple to underlying cause of death (SRMU) for available years between 1999 and 2012 for the following diseases (ICD-10 codes in parenthesis): diabetes (E10-E14), ischemic heart disease (IHD, I20-I25), other heart disease (I00-I09, I30-I33, I39-I45, I47-I48, I49.1-I49.9, I50-I52), cerebrovascular disease (I60-I69), and renal failure (N17-N19). These diseases were chosen because of their high or low rates in Israel compared to other countries, availability of international data from the Multicause Network, and their potential interchangeability as UC of death [12, 13]. We did not investigate other causes which are often comorbid with diabetes such as obesity, Alzheimer’s disease and dementia, and skin diseases, since, according to WHO coding rules, they should not be chosen as UC when listed with diabetes.

Age standardization was to the 2013 European standard population.

The SRMU, CDAI and a 95 % confidence interval for the CDAI were calculated for Israel in 2009 as specified by Desesquelles et al. [3, 4]. A 95 % confidence interval, based on the standardized rates, was also calculated for the SRMU. The mean number of causes per death certificate, (excluding ill-defined causes) was also calculated, as this can be expected to affect the MCOD indicator values.

Israeli results were compared with those from Italy [14], France [15] and the USA [16] for 2009, and Czech data for 2011 [17], available from the Multicause Network [4], and Australian values published for 2007 [5]. We used Czech data for 2011 because it was coded after the introduction of automatic coding with Iris and reported to be more reliable and internationally comparable [18].

There was no need for ethical approval since the data used was unidentified.

## Results

**Fig. 1:**
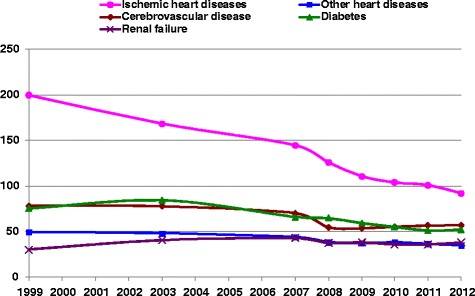
**Age-standardized underlying mortality rates in Israel, by causes of death, 1999–2012.** Rates/100,000 population, age standardized to 2013 European standard population

**Fig. 2:**
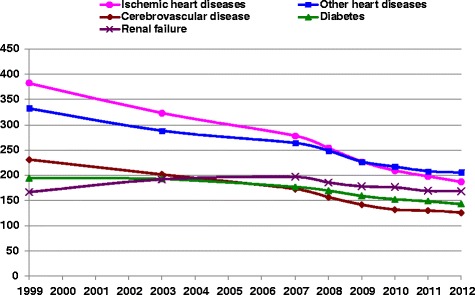
**Age-standardized multiple mortality rates in Israel, by causes of death, 1999–2012.** Rates/100,000 population, age standardized to 2013 European standard population

**Fig. 3:**
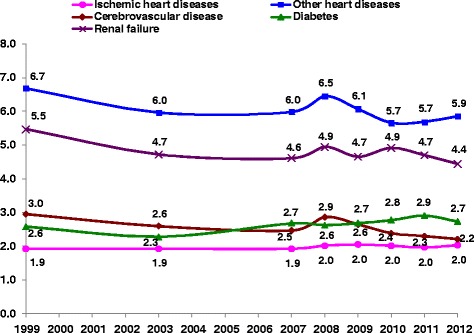
**The Standardized Ratio of Multiple to Underlying mortality rates (SRMU), by causes of death, 1999–2012.** Ratio of rates, age standardized to 2013 European standard population

Figures 1 and 2 show trends in standardized mortality rates as UC and MCOD for diabetes, renal failure, IHD, other heart disease and cerebrovascular diseases. All these diseases, except renal failure, had steadily decreasing rates both as UC and MCOD between 1999 and 2012. The highest decrease, 51 %, was for IHD, 54 % as UC and 51 % as a MCOD between 1999 and 2012. The rate of decrease was similar for diabetes as UC and MCOD, 27 % and 30 % respectively. Cerebrovascular disease and other heart disease showed a greater decrease as MCOD than UC. The renal failure mortality rate increased by 25 % as UC, while as MCOD, after an initial increase, it decreased to the same rate in 2012 as 1999. The SRMU shown in Fig. 3 reflect the relative differences in change between MCOD and UC. For IHD and diabetes the SRMU was relatively stable over the years, although it decreased for all other diseases. The SRMU for diabetes ranged between 2.3 and 2.9, and for cerebrovascular disease started at 3.0 in 1999, but decreased over this period to 2.2 in 2012. The SRMU for renal failure, also decreased by about 20 % from 5.5 to 4.4, while that for other heart disease had a relatively smaller decrease from 6.7 to 5.9.

Table 1 shows, for selected causes of death, the age-standardized mortality rates for underlying and multiple causes, the SRMU and the average number of causes per certificate for each country in this comparison. Israel has the highest average number of causes per certificate and France and the USA the lowest. In France a maximum of 6 causes is allowed per certificate compared to 13 in Italy [3], and 20 in the USA and Australia [5, 6]. In Israel, where the certificates are not automated, the only limit is the space on the form and a maximum of 16 causes was found.

**Table 1:** Age-standardized mortality rates per 100,000 for underlying and multiple cause, Standardized Ratio of Multiple to Underlying cause (SRMU) and average number of causes by country, selected causes of death, 2009^a^

**Cause of death/country**	**France**	**Italy**	**USA**	**Czech Republic**	**Australia** ^**b**^	**Israel**
**Standardized mortality rate for underlying cause (UC)**						
Diabetes	20	33	31	27	18	59
Ischemic heart disease	68	126	181	337	108	111
Other heart disease	74	68	58	70	N/A	37
Cerebrovascular disease	56	104	61	140	55	54
Renal Failure	12	15	20	8	10^c^	38
**Total mortality**	**968**	**986**	**1100**	**1248**	**885** ^**d**^	**920**
**Standardized mortality rate for multiple cause (MC)**						
Diabetes	60	117	106	169	61	159
Ischemic heart disease	109	200	257	551	174	227
Other heart disease	211	297	221	479	N/A	227
Cerebrovascular disease	97	194	102	245	96	142
Renal Failure	68	138	103	83	58	178
**SRMU**						
Diabetes	3.0	3.5	3.4	6.2	3.4	2.7
Ischemic heart disease	1.6	1.6	1.4	1.6	1.6	2.0
Other heart disease	2.9	2.7	3.8	6.8	N/A	6.1
Cerebrovascular disease	1.7	1.9	1.7	1.8	1.8	2.6
Renal Failure	5.6	9.4	5.0	9.9	5.9^c^	4.7
**Average number of causes (without ill defined)**	2.5	3.3	2.6	3.1	3.1	3.5

Source of data: Italy [14], France [15], USA [16], Czech Republic [17]

Australia: Australian institute of Health and Welfare publication [5]

Total mortality – includes all causes of deaths

^a^ Australia – 2007, unstandardized rates, Czech Republic – 2011

^b^ Cause specific mortality rates for Australia are not standardized

^c^ For chronic and unspecified renal failure (N18-N19), and for other countries N17-N19

^d^ Standardized rate for 2009: Based on data downloaded from the Australian institute of Health and Welfare [35]

Age standardized mortality rates for diabetes as UC and MCOD were lowest in France and Australia, and highest in Israel. The rate for IHD was very high in the Czech Republic and in the USA and lowest in France. The mortality rate for cerebrovascular disease was highest in the Czech Republic and also high in Italy. Other heart disease was lowest in Israel, while renal failure was highest in the Czech Republic and lowest in Australia.

The SRMU for diabetes and renal failure was lowest in Israel, 2.7 (95 % CI 2.6–2.8) for diabetes, compared to 3.0 in France, and 3.4–3.5 in Italy, USA and Australia and 6.2 in the Czech Republic. The SRMU for renal failure was very high in Italy and the Czech Republic (9.5, 9.9) compared to Israel, 4.7 (95 % CI 4.4–4.9), and the USA, France and Australia (5.0, 5.6, 5.9). The SRMU for IHD and cerebrovascular disease, 2.0 (95 % CI 2.0–2.1) and 2.6 (95 % CI 2.5–2.8) respectively, were higher in Israel than other countries, particularly for cerebrovascular disease, 2.6 compared to 1.7–1.9. The SRMU for other heart disease in Israel, 6.1 (95 % CI 5.7–6.4), was higher than all countries except the Czech Republic.

Table 2 shows the CDAI indicator for diabetes and renal failure as UC with contributing cause of IHD, other heart disease and cerebrovascular disease. There were significantly high associations of all the cardiovascular causes with diabetes in all countries (95 % CI above 100), except for other heart disease in the Czech Republic and Italy. Values were particularly high for IHD in France and the USA. Renal failure was also significantly associated with IHD and other heart disease in most countries, but not with cerebrovascular disease. In Israel, this association can also be seen from the fact that 40 % of those with diabetes and a quarter of those with renal disease as UC had IHD as a contributory cause. About a third of those with UC of diabetes and renal disease had other heart disease as a contributory cause, and about a quarter of those with UC of diabetes had cerebrovascular disease as a contributory cause.

**Table 2:** The Cause of Death Association Indicator (CDAI), 2009^a^

	**Multiple cause (MC)**		
	**Ischemic heart disease**	**Other heart disease**	**Cerebrovascular disease**
**UC diabetes**			
**France**	450^b^	172^b^	230^b^
**Italy**	296^b^	108^b^	211^b^
**USA**	387^b^	162^b^	262^b^
**Czech Republic**	157^b^	99	149^b^
**Israel**	223^b^	144^b^	228^b^
**Israel : % with MC**	39.8	32.7	24.4
**UC Renal failure**			
**France**	194^b^	183^b^	88
**Italy**	148^b^	118^b^	93
**USA**	140^b^	134^b^	86
**Czech Republic**	130	136^b^	63
**Israel**	143^b^	134^b^	96
**Israel : % with MC**	25.1	31.0	10.1

Source of data: Italy [14], France [15], USA [16], Czech Republic [17]

Formula for CDAI [3] $\mathit{\mathsf{C}}\mathit{\mathsf{D}}\mathit{\mathsf{A}}{\mathit{\mathsf{I}}}_{\mathit{\mathsf{u}},\mathit{\mathsf{c}}}=\frac{{\displaystyle {\sum}_{\mathit{\mathsf{x}}}\frac{{}_{\mathit{\mathsf{u}}}\mathit{\mathsf{d}}_{\mathit{\mathsf{c}},\mathit{\mathsf{x}}}}{{}_{\mathit{\mathsf{u}}}\mathit{\mathsf{d}}_{\mathit{\mathsf{x}}}}.{\overline{\mathit{\mathsf{d}}}}_{\mathit{\mathsf{x}}}}}{{\displaystyle {\sum}_{\mathit{\mathsf{x}}}\frac{{\mathit{\mathsf{d}}}_{\mathit{\mathsf{c}},\mathit{\mathsf{x}}}}{{\mathit{\mathsf{d}}}_{\mathit{\mathsf{x}}}}.{\overline{\mathit{\mathsf{d}}}}_{\mathit{\mathsf{x}}}}}*\mathsf{100}$

${}_{\mathit{\mathsf{u}}}\mathit{\mathsf{d}}_{\mathit{\mathsf{c}},\mathit{\mathsf{x}}}$ = number of deaths observed at age *x* with underlying cause *u* and contributing cause *c*

${}_{\mathit{\mathsf{u}}}\mathit{\mathsf{d}}_{\mathit{\mathsf{x}}}$ = number of deaths observed at age *x* with cause *u* as underlying cause

*d*_*c,x*_ = total number of deaths observed at age *x* with cause *c* as contributing cause (regardless of the underlying cause)

*d*_*x*_ = total number of deaths observed at age *x* (regardless of the underlying cause)

$\overline{{\mathit{\mathsf{d}}}_{\mathit{\mathsf{x}}}}$ = standard number of deaths at age *x* (number of deaths from the 2009 WHO life table for high income countries)

^a^Czech Republic - 2011

^b^ Significantly above 100 with 95 % CI calculated as specified in [4]

## Discussion

The data we presented in Table 1 showed that the total age-adjusted mortality rate in Israel was lower than France, Italy, USA and the Czech Republic and higher than Australia although Israel’s rank varied by cause of death. We presented disease rates for these countries from MCOD and comorbidity indicators, and asked whether they could support the suggestion that differences in coding practice, such as choice of UC of death or physician’s filling in of death certificates leads to this distribution of UC.

### Number of causes listed

The first question in the comparability of MCOD data is how many causes are listed on death certificates. The lowest average number found in France is consistent with the low limit on number of causes in their certificate, but the average number in the USA was similarly low, despite the large number of causes allowed. Rates of mortality from multiple causes listed, and corresponding SRMUs would be expected to be higher in countries where more causes are usually recorded as would be the case for Israel with the highest mean number of causes.

### Diabetes

In the case of diabetes, we found the SRMU in Israel to be lower than other countries despite the higher average number of causes. This would support the suggestion that diabetes is chosen as UC more often. The low SRMU for diabetes is also supported by the high association of diabetes with CVD, frequently mentioned amongst multiple causes, as shown in Table 2 for all countries. In Israel, diabetes as UC was found with IHD (40 % of cases), cerebrovascular disease (33 %) and other heart disease (25 %).

Table 1 shows that the multiple mortality rate from diabetes in Israel was higher than France, the USA, Italy and Australia. Therefore, even if diabetes was chosen less often as UC, as we saw in other Western countries (SRMU = 3.0–3.5), the mortality rate as UC would still be considerably higher than other countries.

It thus appears that the high mortality rate from diabetes is valid, although somewhat higher than it should be by international standards.

Is this supported by prevalence rates of diabetes in Israel? Although crude total diabetes prevalence in Israel has not been reported as exceptionally high, 7.6 % in 2011 for adults aged 20–79, ranking slightly above the OECD average of 6.9 % [19], a careful comparison of recent data shows a somewhat different picture. We received age specific data from the International Diabetes Federation (IDF) from their recently published update of diabetes prevalence in 2014 for European countries. Israeli data was received from the National Program for Quality Indicators in Community Healthcare in Israel for 2014 [20]. This program collects data from Health Funds, which provide universal coverage to all citizens in Israel, and therefore should give an accurate estimate, better than the survey based IDF data. Results by age are shown in Additional file 1: Figure S1).

We found that at young ages, Israel has one of the lowest rates of diabetes prevalence amongst European countries. However, for the intermediate age group of the IDF, 40–59, Israel’s rank increased, and for the oldest age group, 60–79, the prevalence in Israel is among the highest, after Portugal (which had the next highest mortality rate from diabetes amongst European countries [1]). This further validates the high mortality rate.

Why is diabetes mortality so high in Israel? In the last decade, there has been a heightened awareness in the medical community of diabetes and treatment options which may also lead to its frequent mention on death certificates. There may be genetic differences in disease susceptibility and in disease complication rates since the genetic pool in Israel is a unique combination of the many ethnic origins of its inhabitants. Lastly, perhaps our higher rates of mortality from diabetes represent not an over-diagnosis of diabetes- caused death in Israel, but rather an under-diagnosis in other countries. For example, in a USA study of diabetics, diabetes was recorded on only 41 % of the death certificates of decedents, and chosen as UC for 13 %, leading to a considerable underestimate of the burden of disease [21].

It should be noted that the decreasing trend in the mortality rate for diabetes in Israel which we saw in Figs. 1 and 2 between 2003 and 2012 was the same for diabetes as UC and as a MC, and the SRMU was also relatively stable over the period, showing that there have not been significant changes in certifying and coding practice for diabetes in Israel. This is different from the rates reported by Lin at el. in Taiwan [22], where the trend in UC rates was different from the trend in MCOD rates. Lin reported that the USA, like Israel, had similar UC and MCOD trends.

### Renal failure

We saw in Table 1 the high rates of renal failure in Israel both as UC and MCOD. We also saw that the SRMU for renal failure is marginally lower than France, Australia and the USA. Table 2 showed that ischemic and other heart diseases were frequently found as associated causes both in Israel and other countries, but not cerebrovascular disease. Here, too, it could be that renal failure is chosen more frequently as UC than if should be instead of, for example, ischemic and other heart diseases.

Renal failure mortality includes both chronic and acute renal failure. If chronic renal failure mortality is truly high it should be reflected in chronic kidney disease rates. OECD data for the prevalence rate in 2011 of end-stage renal failure in Israel treated by dialysis or kidney transplants does show Israel amongst the highest of OECD countries, although well below that of the USA ([23], Additional file 2: Figure S2). A recent publication by the Israeli Society of Nephrology and Hypertension (ISNH) with the Israeli Center of Disease Control (ICDC) [24] also shows very high incidence rates of new patients receiving renal replacement therapy in 2009 compared to other countries, particularly at ages over 45 when Israeli rates were second highest, with only the USA having higher rates.

Acute renal failure, however, is not connected to end-stage renal disease. A recent WHO published document notes that causes like renal failure are, in fact, “modes of dying” rather than “causes of death” and therefore should be reported with a precipitating cause, and not generally listed as underlying cause at all [25]. A comparison of acute and chronic renal failure mortality rates from the European Detained Mortality Database [26], shows that Israeli mortality rates are particularly high for acute renal failure, about six times that of Western EU members (a computed weighted average), compared to under three times for chronic renal failure. These high rates could be due to the certifying physician incorrectly listing acute renal failure as UC of death.

### Cardiovascular diseases

Table 1 shows that the SRMU for cardiovascular diseases was higher in Israel than other countries implying that they were chosen as UC less often, but also reflecting the higher average number of causes. Replacing diabetes and renal failure with heart diseases as UC, as suggested above, would lead to higher mortality rates for IHD and other heart disease although the rates would still be low relative to other Western countries. Similarly, cerebrovascular disease would replace some of the diabetes mortality, but still remain low. We did note in Fig. 3 a trend of decreasing SRMU for cerebrovascular disease, from 3.0 in 1999 to 2.2 in 2012, nearer to that of other countries, and indicating that it is already being coded more frequently as UC.

### Health policy implications and future trends

#### Diabetes

Although we saw in Figs. 1, 2 and 3 decreasing mortality rates from diabetes in recent years, both as UC and MC, with a relatively stable SRMU, the rates are still well above other Western countries. We showed that some of the excess may be due to differences in choosing UC, but it remains a challenge to the health system in Israel to continue efforts to control this disease, both by treatment and prevention. The recent OECD health care quality review on Israel [27] reported diabetes care to be good and improving in recent years, but noted a particularly high prevalence among certain population groups, such as Israeli-Arabs (constituting 20 % of the population) and Ethiopian immigrants (constituting 1 % of the population), who may have lower quality of care. The OECD review also suggests that Israel should step up efforts to manage diabetes complications and co-morbidities, and improve care co-ordination.

Education in healthy living such as that promoted by the inter-ministerial program for healthy and active living [28], or by initiatives such as that reported by the Clalit Health fund in the Sharon and Shomron area for the Arab population, which organizes culturally attuned programs for healthy living amongst Arab women [29], are very important. We hope that these efforts will lead to a lowering of mortality rates to the level of other countries.

A hopeful trend is noted in a recent paper by Tomas Karpati et al. [30] on the prevalence and incidence rates of diabetes in the Clalit Health Fund, which insures more than half the Israeli population. They reported that although prevalence rates were rising steadily, in the last few years the age standardized prevalence rate and rates at younger ages have stabilized, while the incidence rate actually decreased since 2006. This trend will hopefully also lead to a reduction in mortality from diabetes.

#### Renal failure

Why is renal failure so high in Israel? Since diabetes often leads to end-stage renal failure (ESRF), its high prevalence in Israel at older ages contributes to the high renal failure rates. This is supported by the high proportion of diabetes among new patients receiving renal replacement therapy (RRT) in Israel, which increased from 19 % in 1989 to 41 % in 2001 [30] and 43 % in 2009 [24]. We see from the ISNH report [24] that this proportion was relatively stable over the last decade, as was the incidence rate of new RRT patients, after a steep increase during the 1990’s.

Calderon et al. [31] discuss other reasons for the high rate of ESRF reported in Israel. They note that RRT is provided in Israel to all patients with ESRF with no official age or comorbidity restrictions, and since 1995 the health funds which provide insurance are reimbursed for dialysis treatment. As well as high Israeli rates of incidence and prevalence of chronic renal failure, there may be differences in progression rates from chronic renal failure to ESRF. In addition, a large wave of immigrants came from the Former Soviet Union (FSU) in the 1990’s, who were characterized by a high prevalence of chronic disease [32], including renal disease, and now are about 10 % of the Israeli population. The subsequent decrease in their immigration in the last decade, as well as possible improvements in diabetes and hypertension treatment may have led to the stabilizing of the incidence and prevalence of ESRD, as well as the decreasing trend in renal failure mortality as a MCOD and to a lesser extent as UC, which we saw in Figs. 1 and 2. An improvement in certification practices may lead to a reduction in the coding of acute renal failure as UC.

#### Death certification and coding practice

Our findings support the need for physicians to be trained to fill in death notification forms correctly, following international guidelines on determining the true UC of death, and not the immediate symptoms, to ensure correct international comparability of death mortality data. Although amongst physicians’ routine duties, death certification is not currently part of the medical curriculum, and physicians are expected to learn on their own, using an instructional pamphlet as a reference. The Israel Health Ministry is embarking upon a web based training program that might become compulsory for all medical graduates.

Lu et al. [33] have raised another certification and coding problem which can affect diabetes mortality rates. Incorrect causal sequences on part I of the death certificate, such as those with hypertension reported after diabetes may lead to hypertension being reported as UC instead of diabetes, particularly if coding rules are not applied correctly. This, too, supports the need for the physician training program mentioned above.

The coding of UC in the CBS is done manually by a relatively small number of coders, so mistakes by one coder can have significant and lasting effects on statistics. The CBS administers an internal quality control process in which a sample of one tenth of cases is coded twice each year. A high level of agreement, 91–96 % of cases, was found between 2007 and 2012. The CBS makes efforts to encourage discussion and consultation between coders to ensure uniformity and data valid for international comparisons.

There are plans to automate the notification of death form in Israel. This would limit the number of causes and enable automatic choice of UC according to WHO coding rules, using a computerized system such as ACME (Automated Classification of Medical Entities), which many other countries have implemented. This could redress some of the coding issues we found in this study and make Israeli data more comparable internationally.

Israel has a high proportion of immigrant physicians. In 2013, 37 % of doctors under 65 were born in Eastern Europe [34]. The somewhat limited knowledge of Hebrew and English of these immigrants might also lead to errors in completing the form. Automation plans mentioned above should help this too, but in the meantime, a more user-friendly death notification form with an English translation, might help.

### Limitations

Reference to MCOD data and indicators shows the complexity of choosing the UC and how coding practices can differ among countries. The Czech Republic, in contrast to Israel, rarely codes diabetes and renal failure as UC as seen by the high SMRU. As life expectancy increases and with it increased comorbidities, it becomes harder to choose one UC and therefore international comparisons based on UC only may be misleading.

The first limitation on the use of MCOD data is its availability. It would be useful if more countries provided their MCOD data to a central database such as the MultiCause Network [4]. Another problem is that the indicator values are dependent on the number of causes, which varies between countries. We expect the SRMU to be higher the more causes are listed and the CDAI to be lower. We saw in Table 2 that France and the USA with lowest number of causes in general have higher CDAIs. Numerical comparisons are therefore not possible with the current indicator definitions.

Strict comparability between countries depends on similar data collection and coverage. The highest age group available in Israel for mortality data is 85 and over, while the European Standard Population gives weights for ages up to 95 and over. Grouping ages over 84 into additional groups results in higher age-standardized rates, affecting the comparison with Israel.

## Conclusion

This international comparison suggests that diabetes and renal failure may be coded more frequently in Israel as UC and that they are coded instead of heart and cerebrovascular disease. However, even with coding changes, diabetes and renal failure mortality rates would be high compared to other countries, similar to their high rate as MCOD. The comparatively high diabetes prevalence in Israel at older ages and the high rate of ESRF indicate a need for improvement in prevention and care.

This study highlights the need for training physicians on death certification practice and the importance of further progress towards automation in recording and coding death causes.

## Additional files

Additional file 1:
**International comparison of diabetes prevalence by age (percent of population), 2014.**
Additional file 2:
**International comparison of end stage renal failure prevalence, 2011.**
