# Supplementary material for: High Israeli mortality rates from diabetes and renal failure - Can international comparison of multiple causes of death reflect differences in choice of underlying cause?
Source: Isr J Health Policy Res. 2015 Oct 1;4:31. doi: 10.1186/s13584-015-0027-6 (PMC4590706; doi:10.1186/s13584-015-0027-6)
Supplement: Additional file 1: — International comparison of diabetes prevalence by age (percent of population), 2014. [file 13584_2015_27_MOESM1_ESM.doc]

**International comparison of diabetes prevalence by age (percent of population), 2014**

Source: International Diabetes Federation (IDF) Atlas data, 2014, Israeli data from Quality Indicators in Community Healthcare (QICH), 2013.
