# Supplementary material for: High Israeli mortality rates from diabetes and renal failure - Can international comparison of multiple causes of death reflect differences in choice of underlying cause?
Source: Isr J Health Policy Res. 2015 Oct 1;4:31. doi: 10.1186/s13584-015-0027-6 (PMC4590706; doi:10.1186/s13584-015-0027-6)
Supplement: Additional file 2: — International comparison of end stage renal failure prevalence, 2011. [file 13584_2015_27_MOESM2_ESM.doc]

**International comparison of end stage renal failure prevalence, 2011**

Rates per 100,000 persons. Source: OECD Health Statistics 2014
